# Presence and localization of apelin and its cognate receptor in canine testes using immunohistochemical and RT-PCR techniques

**DOI:** 10.1007/s11259-022-10001-0

**Published:** 2022-11-04

**Authors:** Alessandro Troisi, Cecilia Dall’Aglio, Margherita Maranesi, Riccardo Orlandi, Chiara Suvieri, Sara Pastore, Marilena Bazzano, Marcelo Martínez-Barbitta, Angela Polisca

**Affiliations:** 1 Scuola di Bioscienze e Medicina Veterinaria, Università di Camerino, Via Circonvallazione 93/95, 62024 Matelica (Macerata), Italy; 2 Dipartimento di Medicina Veterinaria, Università di Perugia, Via San Costanzo 4, 06126 Perugia, Italy; 3 Tyrus Clinica Veterinaria, Via Aldo Bartocci, 1G, 05100 Terni, Italy; 4 Dipartimento di Medicina e Chirurgia, Università di Perugia, piazzale Severi 1, 06132 Perugia, Italy

**Keywords:** Adipokines, Adipose tissue, Canine, Germinative cells, Reproduction

## Abstract

Apelin, a member of the adipokine family, is a novel endogenous peptide which regulates the male reproductive system of mammals by interacting with a specific receptor. Recent studies have highlighted that apelin may play a role in the regulation of reproduction by reducing testosterone production and inhibiting LH secretion. To the best of our knowledge, there is no available data on the presence of the apelin and its receptor in canine testes. Therefore, the aim of this study was to reveal the presence of apelin and evaluate its distribution in the canine testes using immunohistochemical and RT-PCR techniques. For this purpose, five fertile and healthy male dogs were subjected to elective orchiectomy. The immunohistochemical reaction revealed the presence of apelin and its receptor in the canine testes. Apelin was localized in spermatids and spermatozoa with a positive signal in the “acrosomal bodies”. As regards the apelin receptor, a positive immunoreaction was detected in the cytoplasm of the cells localized near to the basal membrane of the seminiferous tubules and in the cytoplasm of Leydig cells. The RT-PCR analysis showed the presence of transcripts for apelin and apelin receptor in all of the samples under study. A 35kDa band confirmed apelin receptor protein expression in all of the samples analysed. In conclusion, the paracrine and endocrine role of apelin and its cognate receptor on male reproduction reported in humans and laboratory animals could also be hypothesized in dogs.

## Introduction

Adipose tissue is an active metabolic organ that requires intrinsic systems to maintain its function and health (Ferhat et al. 2019). It is widely known that it produces specific factors called adipokines such as chemerin, visfatin, adiponectin, leptin, resistin and apelin. These molecules can engage in complex communication between adipose tissue and other organs producing autocrine, paracrine or endocrine effects (Vázquez-Vela et al. 2008). The correlation between adipokines and reproduction has been reported in humans and several other species (Shokrollahi et al. 2021). Apelin (APLN) is an endogenous peptide that was originally isolated from bovine stomach extracts (Tatemoto et al. 2001). This pleiotropic molecule fulfills its function by binding to a G protein-coupled receptor (GPCR) called APJ (or APLNR) which is structurally similar to an angiotensin II receptor type 1 (Tatemoto et al. 2001). The peptide precursor of apelin is composed of a chain of 77 amino acids. Through enzymatic catalysis many molecular forms with different biological functions can be obtained from this apelin precursor: apelin-13, apelin-17, apelin-36, and pyroglutamate-apelin-13 (Tatemoto et al. 2001). The shorter forms of apelin have proved to possess greater biological potency than longer forms (Tatemoto et al. 2001). APLN and its receptor have been detected in many tissues of numerous mammalian species. In fact, transcript and protein for both molecules are expressed in specific hypothalamus and cerebroventricular regions of the brain, pituitary gland, skeletal muscle, kidney, spinal cord, thyroid gland, lungs, heart, adipose tissue and reproductive tract (Medhurst et al. 2003; Carpéné et al. 2007; Falcao-Pires et al. 2010; Mercati et al. 2018; Shokrollahi et al. 2022). In veterinary medicine, apelin was isolated from cow and sow ovaries (Roche et al. 2017) and from canine placenta (Troisi et al. 2020). It appears to play an important role in biological events such as folliculogenesis, cellular proliferation or apoptosis and the release of steroid hormones (Estienne et al. 2019). It is also involved in angiogenesis during pregnancy (Troisi et al. 2020). APLN and its receptor have recently been detected in the male gonads of human and laboratory animals (Medhurst et al. 2003; Estienne et al. 2019; Kawamata et al. 2001; Pope et al. 2012). More specifically, it has been demonstrated that the intracerebroventricular infusion of apelin in male rats reduces serum testosterone levels thus causing a drastic reduction in the number of testosterone-producing Leydig cells (Sandal et al. 2015). Therefore, it has been hypothesized that they may have an important role in male reproductive system function. To the best of our knowledge, there is no available data on the presence of apelin and its receptor in domestic animals. Therefore, the aim of this study was to highlight the presence and distribution of apelin and its receptor in canine testes using immunohistochemical and RT-PCR techniques.

## Materials and methods

### Animals

Testicular tissues were collected from 5 healthy mixed breed dogs (aged 2 to 5 years) with an average weight of 30kg. The dogs were admitted to the Veterinary Teaching Hospital (VTH) of the University of Perugia for elective orchiectomy with the written consent of their owners.

### Surgical procedures

The orchiectomy was carried out under general anaesthesia according to the following protocol: premedication with methadone 0,2 − 0,4mg/kg IM (or IV) and medetomidine 1–5 mcg/kg; induction with preoxygenation and propofol 4–6mg/kg IV; anaesthesia for surgery maintained with isoflurane, adjusting the vaporiser setting according to anaesthetic depth; þ IV Ringer 10 ml/kg/h. A conventional surgical technique was used.

### Tissue collection and processing

Following orchiectomy, the testicular tissue samples were promptly removed from all animals and thoroughly washed with saline. After dividing the tissue samples into small pieces under stereoscopic magnification, they were immediately sent for examination. For the RT-PCR, the samples were rinsed with RNase-free water then immediately frozen at -80C and were later evaluated for gene and protein expression. For the immunohistochemical analysis, some small pieces of testicular tissue were fixed by immersion in 4% (w/v) formaldehyde in PBS (pH 7.4) for 24h at room temperature and then processed following routine tissue preparation procedures (Dall’Aglio et al. 2014).

### RT-PCR

#### Reagents

Deoxyribonuclease I (DNAse I Amp. Grade), Superscript III Reverse Transcriptase (Superscript III First-Strand Synthesis System) and DNA ladders were purchased from Life Technologies Italia (Monza, Monza Brianza, Italy). Reagents for the isolation and purification of total RNA (TRIzol), Taq DNA polymerase (Platinum), RNAse free tubes, water and deoxyNTPs, and primers for APLN and APLNR were also purchased from Life Technologies while NucleoSpin Gel and PCR clean up were supplied by Macherey-Nagel Inc (Bethlehem, PA, USA).

#### APLN and APLNR gene expression qualitative analysis

Total RNA was extracted from the testicular tissues (100mg each) of five male dogs. DNAse treatment was performed following the manufacturer’s instructions. Five micrograms of total RNA were reverse transcribed in 20 µL of Superscript III First-Strand Synthesis System using random hexamer according to the protocol provided by the manufacturer. The polymerase chain reaction (PCR) method without reverse transcriptase was used to test for genomic DNA contamination. The multiplex PCR amplification was performed as previously described (Mercati et al. 2019) using 1.0 µL of complementary DNA as a template for *APLN* and *APLNR* primers (Table1). Cycling conditions consisted of an initial denaturalizing cycle at 94C for 75s followed by 35 cycles for each target gene at 94C for 15s, 60C for 30s, 72C for 45s and a final extension step at 72C for 10min. For each experiment the complete set of samples was processed in parallel in a single PCR using aliquots of the same PCR master mix. The amplified PCR-generated products (18 µL of 25 µL total reaction volume) were analysed by electrophoresis on a 2% agarose gel using ethidium bromide staining (Troisi et al. 2020). The amplified products, collected from the agarose gel following electrophoresis, were purified with a Nucleospin Extract II kit and their identity was confirmed by DNA sequencing using the Sanger technique.

**Table 1:** *APLN/APLNR Primers (Canis lupus familiaris) used for RT-PCR (*Troisi et al. 2020)

Gene	Gene bank accession n.	Product region		Primers	bp
***APLN***	XM_849169.5	589–759	F	CCTCCTGCAACTCTGGCTAC	171
			R	GTGGGAGACAAAGGGAATCA	
***APLNR***	XM_005631209.3	1505–1602	F	AGTCAGGTAGCATGACAGCAC	97
			R	AGCCTCAAGAAGGAAGGAAGAC	

### Immunohistochemistry and western blot reagents

For the immunohistochemical analysis (IHC), the rabbit polyclonal anti-APLN antibody (NBP2-31176) was purchased from Novus Biologicals (Novus Biologicals, USA); the mouse monoclonal anti-APLNR antibody (sc-517,300) used for IHC and Western Blot analysis was supplied by Santa Cruz Biotechnology (Santa Cruz, CA, USA); the normal goat serum (s-1000), the two secondary biotin-conjugated antibodies, the goat anti-mouse (BA-9200 and goat anti-rabbit (BA-1000) and the ABC Kit and DAB were purchased from Vector Laboratories (Vector Laboratories, Burlingame, CA, USA). Finally, the Eukitt (03989) was supplied by Sigma-Aldrich.

### Immunohistochemistry

Following microwave oven antigen retrieval and using 10 mM citric acid, pH 6.0, (three 5-minute cycles at 750W), five-µm-thick serial sections were placed on poly-lysine-coated glass slides and processed for immunohistochemical reaction,. All subsequent steps were carried out in a moist chamber at room temperature to prevent evaporation of the reagents. After proper cooling the sections were pre-incubated for 30min with specific normal goat serum (1:10) in order to avoid non-specific binding of the primary antibodies. The serial sections were then incubated overnight with anti-APLN rabbit polyclonal (1:100) and anti-APLNR mouse monoclonal (1:100) primary antibodies. The next day, after washing in PBS, the sections were incubated with specific secondary biotin-conjugated antibodies (a goat anti-rabbit and a goat anti-mouse respectively (both 1:200) for 30min). Then, after another washing in PBS using the ABC KIT, the sections were incubated again for another 30min. Finally, the tissue sections were rinsed in PBS and the reaction was developed using diaminobenzidine (DAB) as the chromogen. At the end of the immunoreaction, the sections were rinsed in PBS, counterstained with haematoxylin, dehydrated and mounted in Eukitt. The sections in which the primary antibodies were omitted were used as negative controls of unspecific staining. A canine placenta was used as a positive control for the apelinergic system (Troisi et al. 2020). In order to further confirm the specificity of the anti-apelin receptor antibody, a western blot was performed using the same antibody on testicular tissue. All tissue analyses were carried out on coded slides using a light microscope (Nikon Eclipse E800) connected to a digital camera (Dxm 1200 Nikon digital camera). An image analysis system was used to process the images. The settings for image capture were standardized by subtracting the background signals obtained from the matched tissue sections which had not reacted with the primary antibodies and which were used as immunohistochemical controls (Dall’Aglio et al. 2012). However, these controls were not quantified given the predominantly qualitative nature of the immunohistochemical technique.

### Western blot

The protein expression of APLNR was analysed by WB in all of the testicular samples. In short, total testicular proteins were extracted from the testicular tissue of each dog as previously described (Maranesi et al. 2021). The testicular tissues were homogenized in 300 ml of ice-cold RIPA buffer (PBS containing 1% Igepal CA-630, 0.5% sodium deoxycholate and 0.1% SDS) containing a protease inhibitor (Roche Complete™ Protease Inhibitor Cocktail) under agitation for 2 hat 4°C. Following incubation at 4°C for 20min, the homogenates were centrifuged at 12,000g for 60min at 4°C. The protein concentrations of the supernatants were measured using the bovine serum albumin (BSA) protein assay as reference standard. Equivalent amounts of protein (50mg) were separated by discontinuous 12% SDS-PAGE with 4% stacking gel for 40min at 200V and 500 mA. The proteins were then transferred onto nitrocellulose membrane using the Trans-Blot Turbo System (Bio-Rad). The membrane was then blocked by incubating it in Tris-buffered saline (TBS) containing 0.05% Tween 20 and 3% BSA. Immunoblotting was performed via overnight anti-APLNR monoclonal antibody (1:1000) incubation at 4°C. The membrane was then probed with HRP-labeled anti-mouse IgG antibody (1:10,000) for 60min at room temperature respectively under gentle agitation. All antibody incubations were performed in TBS containing 5% non-fat dried milk and 0.05% Tween-20 (Maranesi et al. 2019). The immunocomplexes were detected using an enhanced chemiluminescence method according to the manufacturer’s protocol (Clarity Western ECL Substrate, Bio-Rad). The blot images were scanned and acquired.

## Results

### RT-PCR

**Fig. 1:**
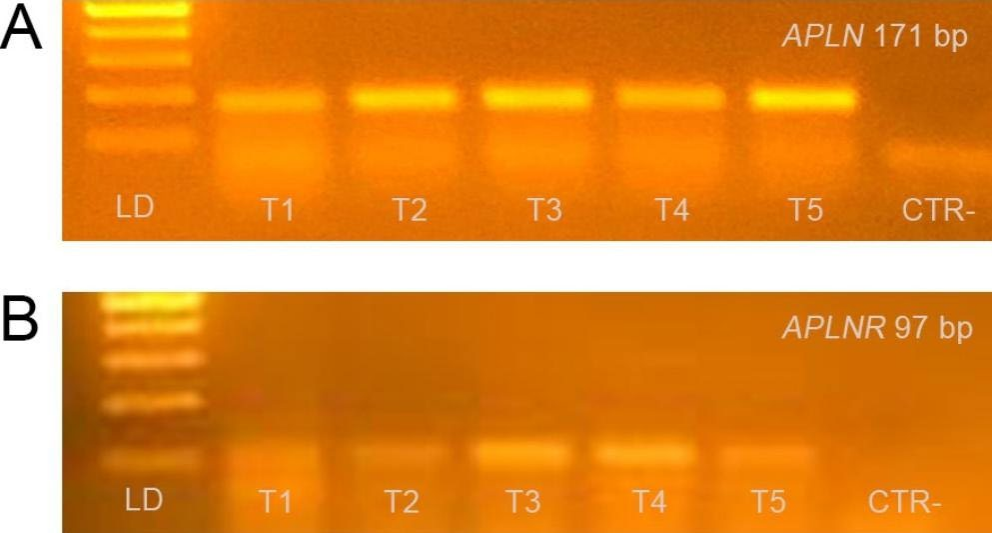
Gene expression of *APLN* (panel A) and *APLNR* mRNA (panel B) in the testicular tissue (T) of five dogs. Representative agarose gel electrophoresis stained with ethidium bromide was used to verify matches between expected and obtained PCR products. Lane CTR– represents a negative control of non-reverse-transcribed RNA submitted to PCR amplification, LD = 100bp DNA ladder

*APLN* and *APLNR* transcripts were detected in all samples. The *APLN* and *APLNR* PCR products were of the expected size (171 and 97bp, Fig.1A and B, respectively).

### Immunohistochemistry

**Fig. 2:**
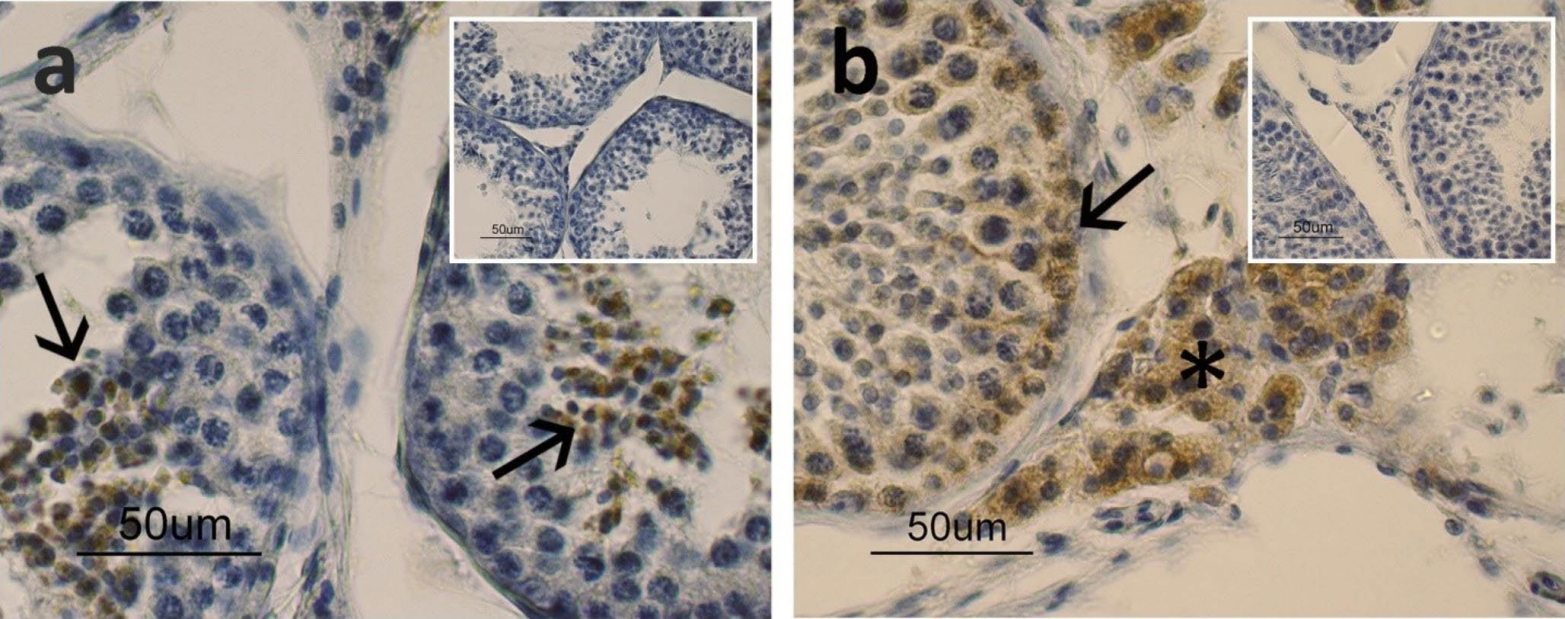
Immunostaining for apelin (a) and apelin receptor (b) in the canine testes. Apelin (a) stained the residual bodies in the apical portion of the seminiferous portions (arrows). The apelin receptor (b) stained the basal line of spermatogenic epithelium (arrow) and Leydig cells in the connective tissue (asterisk). The image insets show the results of the negative controls performed to validate the IHC.

The immunohistochemical reaction showed the presence of apelin and its receptor in the canine testes. Apelin proved to be localized in the apical portion of the seminiferous tubules with a positive signal in the “residual bodies” (arrows, Fig.2a). As regards the apelin receptor, a positive immunoreaction was observed in the cytoplasm of the cells localized in the basal portion of the seminiferous tubules (arrow) and in the cytoplasm of Leydig cells (asterisk). (Fig. 2b)

### Western blot

**Fig. 3:**
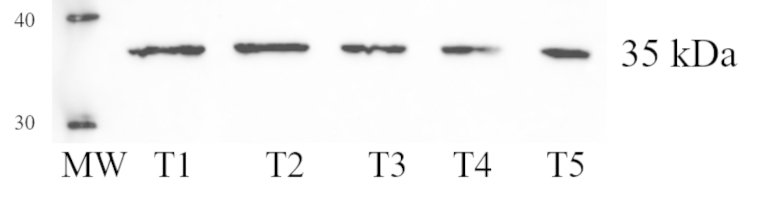
Protein expression in the testicular tissue (T) of five dogs. MW = molecular weight marker

The APLNR protein expression was evaluated with western blot in all of the samples. The immunoblot showed a strong band at approximately 35 Da (Fig.3).

## Discussion

In humans adipokines regulate numerous metabolic processes (Bertrand et al. 2015), including female and male reproductive systems, (Dupont et al. 2015) by controlling the activity of hypothalamic-pituitary gonadal axis under both normal and pathological conditions (Campos et al. 2008). In particular, in mammals, numerous other adipokines directly are able to influence male spermatogenic function (Milon et al. 2019; Kotula-Balak et al. 2022) reported that in landrace boar, immunocastration results in altered adipokine and leptin system. Moreover, some authors report that the change in adipokine expression and their receptor in testis are correlated with senescence and reproductive pathology (cryptorchidism and testis tumors) (Duliban et al. 2020) 0; Ramisz et al. 2021).

The role of apelin and its receptor in animal reproduction has been widely reported (Dupont et al. 2015; Estienne et al. 2019; Roche et al. 2017; Shokrollahi et al. 2021; Troisi et al. 2020). However, to the best of our knowledge, to date few studies have investigated the role that apelin and apelin receptors play in the male reproductive system (Bertrand et al. 2015; Dupont et al. 2015; Campos et al. 2008). Our results revealed, for the first time, the presence of apelin and its receptor in dog testes which are in agreement with those obtained for humans and mice (Medhurst et al. 2003). In particular, this study concerned the immunohistochemical localization of apelin in the residual bodies, in the apical portion of the seminiferous tubules, and of apelin receptor in the cytoplasm of cells localized in the basal portion of the seminiferous tubules. These observations suggest that apelin is involved in spermatogenesis of dog, as well as other adipokines reported in mammalian, via paracrine or endocrine manner mechanism (Thomas et al. 2013; Kurowska et al. 2018). Recently it was observed that the apelin signaling pathway was the most dominant in the seminal plasma of boar (Fraser et al. 2021). This observation could allow us to hypothesize that, if its presence in seminal plasma is confirmed also in the dog, apelin produced by the testes could represent one of the components of the seminal plasma with potential roles in key reproductive events (Martinez et al. 2020).

Despite the study conducted on rats (Brzoskwinia et al. 2020) where both apelin and its receptor were detected in the Leydig cells, in our study only APLNR was found in the cytoplasm of Leydig cells, allowing us to hypothesize the action of the molecule on the control of steroidogenesis with only an endocrine mechanism in dogs.

In conclusion, our results obtained using RT-PCR and immunohistochemical techniques to analyze the dog testis reveal, for the first time, the presence of the APLN-APLNR system in the dog testes under study as previously reported in the testes of adult rats (Brzoskwinia et al. 2020).

These results could provide valuable insights to study the possible role of the apelin/APJ system under normal and pathological conditions in dogs, which could represent an alternative animal model for comparative pathology in addition to the rats and mice commonly used in studies for comparative pathology.

## Data Availability

not applicable.
