# Correction to: Presence and localization of apelin and its cognate receptor in canine testes using immunohistochemical and RT-PCR techniques

**DOI:** 10.1007/s11259-023-10085-2

**Published:** 2023-02-17

**Authors:** Alessandro Troisi, Cecilia Dall’Aglio, Margherita Maranesi, Riccardo Orlandi, Chiara Suvieri, Sara Pastore, Marilena Bazzano, Marcelo Martínez-Barbitta, Angela Polisca

**Affiliations:** 1 Scuola di Bioscienze e Medicina Veterinaria, Università di Camerino, Via Circonvallazione 93/95, 62024 Matelica (Macerata), Italy; 2 Dipartimento di Medicina Veterinaria, Università di Perugia, Via San Costanzo 4, 06126 Perugia, Italy; 3 Tyrus Clinica Veterinaria, Via Aldo Bartocci, 1G, 05100 Terni, Italy; 4 Dipartimento di Medicina e Chirurgia, Università di Perugia, piazzale Severi 1, 06132 Perugia, Italy

**Correction to: Veterinary Research Communications**

10.1007/s11259-022-10001-0

The name of Dr. Marcelo Martínez-Barbitta has been eliminated from the author list, due to an error in the review phase.

The correct list of authors is given below:

- Alessandro Troisi^1^, Cecilia Dall’Aglio^2^, Margherita Maranesi^2^, Riccardo Orlandi^3^, Chiara Suvieri^4^, Sara Pastore^2^, Marilena Bazzano^1^, Marcelo Martínez-Barbitta^2^, Angela Polisca^2^

Also with regard to the contribution of the authors, we note the inclusion of Dr Marcelo Martínez-Barbitta as follows:

- Polisca, Orlandi, Pastore, Martínez-Barbitta and Troisi examined the animals and collected the samples;
  Dall’Aglio carried out the immunohistochemical studies;
  Maranesi and Suvieri performed the RT-PCR and WB studies;
  Troisi, Dall’Aglio, Maranesi and Polisca prepared the manuscript;
  Bazzano analyzed the results;
  Polisca designed and supervised study.

The original article has been corrected.

